# Diet Quality of Older African Americans: Impact of Knowledge and Perceived Threat of COVID-19

**DOI:** 10.3390/ijerph20075274

**Published:** 2023-03-27

**Authors:** Lucy W. Kibe, Mohsen Bazargan, Adaobi Bosah, Katrina M. Schrode, Yufu Kuo, Edward Andikrah, Magda Shaheen

**Affiliations:** 1 Physician Assistant Program, Charles R. Drew University of Medicine and Science (CDU), Los Angeles, CA 90059, USA; 2 Department of Family Medicine, Charles R. Drew University of Medicine and Science (CDU), Los Angeles, CA 90059, USA; 3 Department of Family Medicine, University of California Los Angeles (UCLA), Los Angeles, CA 90095, USA; 4 Department of Psychiatry, Charles R. Drew University of Medicine and Science (CDU), Los Angeles, CA 90059, USA; 5 Department of Internal Medicine, Charles R. Drew University of Medicine and Science (CDU), Los Angeles, CA 90059, USA

**Keywords:** diet quality, COVID-19, older adults, African American, food insecurity, food environment

## Abstract

Confusing health messages and environmental changes intended to prevent the spread of the COVID-19 virus have affected the dietary behavior of older African Americans. We investigated the impact of COVID-19-related factors on diet quality and the relationship between food access and diet quality. We surveyed 150 African Americans aged 55 years and above during the COVID-19 pandemic. The data obtained included socio-demographic and health information, and COVID-19-related knowledge and perceptions. Dietary intake data was obtained using the Diet History Questionnaire III. Analyses included bivariate and multivariable statistics. Overall, based on United States Department of Agriculture guidelines, the diet quality of older African Americans was poor. Lower knowledge and a lower perceived threat of COVID-19 were significantly associated with poor diet quality. Additionally, older African Americans with chronic diseases and food insecurity had poor diet quality. The COVID-19 pandemic has highlighted the fragility of diet quality. The combined impact of poor knowledge and perceived threat of COVID-19, chronic disease, and food insecurity contribute to poor diet quality in this population. This study adds to the well-known need for strategies to support the right to a healthy diet, particularly during COVID-19 and future pandemics. Proactive interventions to counteract the potential consequences of poor diets are needed.

## 1. Introduction

Older African Americans (AA) are disproportionately burdened by morbidity and mortality from the leading causes of death in the US, including cardiovascular disease, diabetes, hypertension, and cancer [1]. Although the importance of a healthy diet for disease prevention, healthy aging, and longevity is well documented [2], many older African Americans fail to adequately meet dietary guidelines based on expert recommendations [3,4,5,6,7]. Dietary behavior during the COVID-19 pandemic was further complicated by confusing health messages, which led to wavering knowledge of COVID-19 and varying perceptions of the threat of the pandemic. Although the severity of the pandemic has eased, poor dietary intake during the pandemic could contribute to long-term detrimental health effects among older adults.

The significance of accurate, unified, and timely public health messages was clearly demonstrated during the pandemic [8,9,10,11,12]. Mixed messages about the severity, risk of transmission, prevention, and treatment of COVID-19 left many worried and unsure which messages were scientifically sound and which were myths [9,11]. In many AA communities, access to testing and eventually vaccines was delayed. These delays, coupled with the disproportionate rate of COVID-19 infections and deaths among AA, may have increased the perceived threat of COVID-19 [9]. Moreover, COVID-19 vaccine hesitancy [13] and general negative aptitudes/mistrust belief toward the healthcare system [14] have been reported among AAs. Grounded in the health belief model (HBM), individuals’ perceptions of their susceptibility to COVID-19 illness, the severity of risk, the potential benefits of health actions in preventing susceptibility, and physical, psychological, financial, and other barriers related to COVID-19 prompted them to adopt healthy or unhealthy dietary behaviors.

In addition to individual behavioral factors, external factors influenced dietary choices and access to adequate and healthy foods during the pandemic. Growing evidence indicates that restrictions to free movement negatively affected peoples’ dietary behaviors [15,16,17,18]. These mandates disrupted the availability and access to quality foods via multiple pathways, including food shortages, shopping limitations, rising costs of commodities, reduced workforce, and financial constraints [19]. These social and economic limitations put a strain on the food environment and compounded the burden of food insecurity. The greatest impact of these changes was felt by older people, low-income individuals, and minorities [20,21,22,23,24,25,26,27]. Among older adults, food insecurity was also associated with higher psychological distress [25,26] and vulnerability to COVID-19 infection [23].

The purpose of this study was to assess the impact of knowledge and the perceived threat of COVID-19 on diet quality among older African Americans. Additionally, we investigated the association between the food environment, food insecurity, and diet quality during the COVID-19 pandemic.

## 2. Methods

***Participants*:** We analyzed data from the COVID-19, Food Insecurity, Exercise and Diet (C-FED) study. The C-FED study was a cross-sectional ‘add-on’ component to a larger intervention among older African American parishioners living in an underserved and under-resourced area of South Los Angeles [28,29]. Participants were eligible for recruitment if they were African American adults aged 65 years or older, or aged 55–64 years with a chronic medical condition. Informed consent was obtained prior to participation. Data was collected by trained interviewers. Interviews were conducted in intervals and took 3–5 h per participant. The study was approved by the Charles R. Drew University of Medicine and Science IRB.

***Measures*:** Study surveys were completed variously through online links, by telephone, or in person with study staff. Data obtained included socio-demographic and health information, COVID-19 perceptions and knowledge, and three questions about the local food environment [30]. We also obtained dietary intake data using the Diet History Questionnaire (DHQ) III. This instrument has been validated using 24 h recalls from the National Health and Nutrition Examination Survey (NHANES) and biomarker studies [31,32]. The DHQ III consists of 135 food and beverage items and 26 dietary supplements, and participants are required to answer questions regarding the type, frequency, and serving sizes of foods, beverages, and dietary supplements consumed within the previous 12 months. The questionnaire allows for separate scores for various nutrients and food groups, as well as an overall food quality score (Healthy Eating Index-2015: HEI-2015). Detailed analytical instructions and coding are provided by the NCI to analyze the DHQ III [33]. Food insecurity was measured using the validated six-item short form of the food security survey developed by the National Center for Health Statistics [34]. Participation in the DHQ III and food insecurity surveys was optional.

### 2.1. Outcome Variable

***Healthy eating index*:** The HEI-2015 (hereafter HEI) was computed based on responses to the DHQ. The HEI is a measure of diet quality based on how closely the diet aligns with governmental recommendations. The total score is calculated based on all foods on a scale ranging from 0–100, with 100 being perfectly aligned with the recommendations. We categorized the subscale variables based on the grading scheme suggested by the USDA, where 90–100 = A; 80–89 = B; 70–79 = C; 60–69 = D; and <60 = F. Due to the small numbers in some categories, we combined grades, so that A–C = moderate–high quality and D–F = fair–poor quality.

### 2.2. Independent Variables

***COVID-19-related constructs*:** Participants responded to questions about their knowledge of COVID-19, their perceived threat of COVID-19, and attitudes toward COVID-19 vaccination using multiple items adopted from the PhenX COVID-19 library [35]. The PhenX (consensus measures for Phenotypes and eXposures) Toolkit is a web-based, publicly available catalog of recommended measurement protocols of phenotypes and exposures [36,37].

***Attitudes toward COVID-19 vaccination*:** This variable was measured using 10 items adopted from the Sage Vaccine Hesitancy Report. Participants were asked to report whether they strongly disagreed, disagreed, neither disagreed nor agreed, agreed, or strongly agreed with statements that were indicative of their attitudes toward the COVID-19 vaccination. A total score was calculated as the average score of the items, ranging from 1: very negative to 5: very positive.

***Knowledge of COVID-19*:** These consisted of 21 true/false items with themes related to symptoms, how the virus is spread, and methods of protection from infection. A total score was calculated. A high score was indicative of greater COVID-19 knowledge.

***Perceived Threat of Infection (risk) of COVID-19*:** Using a scale of zero to seven, participants were asked their agreement with the following statements: (1) thinking about the coronavirus (COVID-19) makes me feel threatened; (2) I am afraid of the coronavirus (COVID-19); (3) I am worried that I or people I love will get sick from the coronavirus (COVID-19); (4) I am stressed around other people because I worry I will catch the coronavirus (COVID-19); and (5) I have tried hard to avoid other people because I do not want to get sick. The average of these scores computed the “perceived threat” score. A high score was indicative of a greater perceived threat of COVID-19.

### 2.3. Other Variables

***Food Access*:** This was measured using questions related to food insecurity and food environment adopted from the USDA food and nutrition service [38]. To avoid multi-collinearity between the food access items, principal component analysis was used to identify potential factors underlying these items. Orthogonal rotation produced two distinct factors, explaining over 79% of the variance. The first factor explained 40% of the variance while the second factor explained 39% of the variance. All items had primary loadings over 0.673, and no item had a cross-loading above 0.235.

***Food Insecurity*:** The first factor produced by the orthogonal rotation was associated with four of the six items that measured food insecurity. The Cronbach’s alpha coefficient for these four items was calculated to be 0.810. A higher score on this index reflects a higher level of food insecurity. This factor is associated with following items: In the past 12 months, (1) I/We could not afford to eat balanced meals; (2) Did you/other adults in your household ever cut the size of your meals or skip meals because there wasn’t enough money for food?; (3) Did you ever eat less than you felt you should because there wasn’t enough money to buy food?; and (4) Were you ever hungry but didn’t eat because you couldn’t afford enough food? Responses to (1) and (2) were often true, sometimes true, never true, don’t know or refused. Responses to (3) and (4) were yes, no, don’t know or refused.

***Food Environment*:** The second factor was associated with the 3 items that measure food environment. The Cronbach’s alpha coefficient for these 3 items was calculated to be 0.962. This factor is associated with the following items: (1) The fresh fruits and vegetables in my neighborhood are of high quality; (2) A large selection of fresh fruits and vegetables is available in my neighborhood; and (3) A large selection of low-fat products is available in my neighborhood. Responses choices were on a 5-point Likert scale, ranging from strongly agree to strongly disagree. A higher score on this index reflects a higher level of perceived environmental food difficulty.

***Health-Related Variables*:** Participants reported their general physical health (good/fair/poor), diagnosed chronic conditions, and COVID-19 vaccine status.

***Socio-Demographic Variables*:** Participants reported their age, gender, level of education, living arrangements, and insurance status. We did not include a measure of income in the analyses because it was strongly correlated with food insecurity.

### 2.4. Data Analysis

We present participants’ descriptive statistics and bivariate relationships between diet quality (HEI), COVID-19 knowledge and perceived threat, health status, and socio-demographic variables. HEI was normally distributed, so we used *t*-tests and Pearson correlations to test for significant associations. We used multivariable linear regression to examine associations of dietary quality and knowledge and perceived threat of COVID-19, controlling for potential confounding factors: food environment and food insecurity orthogonal factors, and other health and socio-demographic factors. Analyses were conducted in SPSS 27 and SAS 9.4. A *p*-value < 0.05 was considered statistically significant.

## 3. Results

Overall, 150 participants completed the baseline surveys. Of these, 118 participants also completed the DHQ III and food insecurity surveys and are included in this analysis (Table 1). Most (70%) participants were female. The mean age was 69 (SD: 9), and almost a quarter were 75 years or older. Almost 14% of the participants had never completed high school. One out of three lived alone, and 98% had health insurance. Over 35% of the participants self-reported their physical health as poor or fair. The average number of chronic conditions was two. More than half and more than one-third of the participants had at least two or at least three medical conditions, respectively. The most prevalent diseases were hypertension (59%), COPD or asthma (24%), diabetes mellitus (22%), and heart disease (11%). The mean HEI was 67.41 (SD: 8.92), which would earn a ‘D’ using USDA suggested scoring.

***In the bivariate analysis,*** diet quality was significantly associated with the perceived threat of COVID-19 (r = 0.23, *p* = 0.016) but not with knowledge of COVID-19 (r = 0.16, *p* = 0.086). Food insecurity was negatively correlated with HEI (r = −0.21, *p* = 0.028), but food environment was not (r = 0.04, *p* = 0.678). The only demographic variable that showed a significant bivariate association with diet quality was gender, where women had a better diet quality (higher HEI) than men (68.6 vs. 64.6; *p* = 0.022). Age, level of education, living arrangements, financial strain, health insurance, physical health status, and COVID-19 vaccination status were not significantly associated with HEI.

***Multivariate Analysis*:** After controlling for confounding variables, both increased knowledge of COVID-19 (*p* = 0.010) and perceived threat of COVID-19 (*p* = 0.004) were positively and significantly associated with diet quality (Table 2). Food insecurity and the number of chronic conditions were negatively associated with diet quality (*p* = 0.042 and *p* = 0.033, respectively).

## 4. Discussion

Given that poor dietary intake is associated with long-term detrimental health effects, especially among older adults, our study investigated the dietary habits of older adults during the COVID-19 pandemic. We explored the impact of knowledge and the perceived threat of COVID-19 on diet quality. We also explored the impact of food access on diet quality during the pandemic. To our knowledge, this is the first study to report these relationships among underserved, older AA adults during the COVID-19 pandemic.

Our study shows that the diets of underserved African American older adults were generally of fair to poor quality. According to USDA diet quality scoring, the mean score was a ‘D’. Due to mandated restrictions and personal fears, many Americans reported changes in food expenditures, shopping behaviors, and increases in online grocery shopping [39]. The ideal way to obtain food during the pandemic was by delivery, which necessitated the use of the internet or ordering apps, methods that may not be ideal for many older adults and underserved communities [40]. For the elderly, barriers to using these innovative methods include inadequate internet connectivity, poorly performing devices, inadequate technology literacy, and dissatisfaction with the quality of food items [40,41,42]. Additionally, food shortages, especially of fresh fruits and vegetables, may have obligated many families to adjust their diets [43,44], with more families opting for food with longer shelf-lives [42]. Other factors contributing to poor quality diet include overall financial constraints due to loss of jobs, limited financial resources to stockpile food, more people to feed at home, inability to participate in food pantries and/or congregate meals, and social isolation [39,45,46].

Consistent with the HBM, individuals who perceived COVID-19 as a threat were more likely to have a higher quality diet. Those with higher knowledge of COVID-19 had a better diet quality. These positive relationships were independent of other factors that could potentially mediate these effects, such as gender, living status, education, and food insecurity, suggesting that individual dietary behavior played a greater role than external factors. A systematic review of 38 international articles showed that the COVID-19 pandemic led to reductions in household food waste, improved cooking behaviors, and food consumption [47]. Other studies evaluating the effect of knowledge and/or perceived threat of COVID-19 found similar positive relationships with the adaptation of protective measures such as handwashing, social distancing, and hygiene rules [8,48,49,50]. Therefore, it is plausible that perception of the harm of COVID-19 and knowledge of associated risks and protective measures prompted the adoption of healthy behaviors, including the consumption of a healthy diet that contains nutrients reported to boost immunity and prevent inflammation such as vitamins B12, C, D, zinc, and iron [51,52,53].

In our study, food insecurity, but not the food environment, was associated with diet quality. Similar to our study, food insecurity has been widely reported among older adults [27] and is disproportionately high among racial/ethnic minority groups [24,54]. Examining the impact of COVID-19 shutdowns on food insecurity among predominantly African American adults residing in an under-resourced community, Dubowitz and colleagues revealed that, despite steady declines since 2011, food insecurity increased from 21% in 2018 to 36% in 2020 and soared to 80% as a result of the pandemic [24]. Our finding of the lack of an association between diet quality and the food environment suggests that the mere presence of quality foods available in the neighborhood does not translate to healthy diets. Therefore, having healthy food available in the neighborhood but in the setting of individuals’ food insecurity is futile. More research is needed to understand the intersection of food insecurity, food environment, and diet quality in order to facilitate effective, community-centered intervention strategies.

Our study shows that the diets of the underserved African American older adults, particularly those with chronic conditions, do not meet healthy eating guidelines. In our study, participants with more chronic conditions had poorer diet quality, further increasing their health risks. Indeed, it has been well-established that among older adults, underserved African Americans are at a greater risk for suboptimal diets and chronic health conditions than their Caucasian counterparts [5,55]. It is worth noting that only 14.7% of our sample reported no chronic conditions. More than half and around one-third reported having at least two or three chronic conditions, respectively. Cardiovascular disease risk factors such as hypertension and diabetes were prevalent. Considering that underserved African American older adults have poor diet quality, food insecurity, and an overabundance of chronic conditions, intervention strategies are needed to address the needs of this group. Health policy discussions should focus on positive and accurate health messages, eliminating food insecurity, and fair distribution of health resources. Special focus should be on those living with chronic conditions. Chatters and colleagues [56] contend that racism and ageism together shape higher risks for coronavirus exposure, COVID-19 disease, and poor health outcomes for underserved older African Americans. If not mitigated, the effects of dietary changes during the COVID-19 pandemic may contribute to poor health outcomes in the future.

**Limitations:** This study had some limitations. The study utilized a convenience sample which could limit its generalizability. The small sample size also limits our confidence regarding the generalizability of the findings, particularly the non-significant associations. However, despite the small sample size, data collection by trained interviewers was rigorous, taking 3–5 h for each participant. Additionally, due to the cross-sectional design, the diet quality of the participants prior to the pandemic is unknown.

## 5. Conclusions

In addition to physiological changes during aging, periods of poor dietary intake can have long-term detrimental health effects. Therefore, interventions are needed, particularly for older underserved African Americans with chronic diseases, to counteract the effects of poor-quality diets induced by COVID-19.

## Figures and Tables

**Table 1:** Characteristics of study participants (n = 118).

Categorical Variables	N (%)
**Age**	
55–64	40 (33.9)
65–74	53 (44.9)
75 and older	25 (21.2)
**Gender**	
Male	36 (30.5)
Female	82 (69.5)
**Education**	
Less than high school degree	16 (13.6)
High school degree	28 (23.7)
Some college	48 (40.7)
Bachelor’s degree	12 (10.2)
Master’s or doctorate degree	14 (11.9)
**Living arrangements**	
Lives alone	40 (33.9)
Does not live alone	78 (66.1)
**Has insurance**	
Yes	116 (98.3)
No	2 (1.7)
**Physical health**	
Excellent/very good	33 (26.5)
Good	42 (36.2)
Fair/poor	41 (35.4)
**Chronic conditions**	
Hypertension	69 (59.5)
COPD or asthma	28 (24.1)
Diabetes	26 (22.4)
Heart disease	11 (9.5)
**At least 2 conditions**	67 (57.7)
**At least 3 conditions**	34 (29.3)
**Fully vaccinated for COVID-19**	
Yes	99 (83.9)
No	19 (16.1)
**Any level of food insecurity**	
Yes	86 (26.5)
No	31 (73.5)
**Continuous variables**	**Mean (SD)**
**Age**	68.52 (8.7)
Number of chronic conditions	1.99 (1.42)
Diet Quality (HEI) (range = 0–100)	67.41 (8.92)
Knowledge of COVID-19 (range 0–2)	1.50 (0.39)
Perceived threat of COVID-19 (range 0–7)	3.54 (2.17)
Attitudes toward COVID-19 vaccination (range 1–5)	4.04 (0.77)
Average raw score for food environment variables (range 1–5)	3.75 (1.06)

**Table 2:** Associations with dietary quality: results of multivariable regression.

Variable/Index	Standardized Coefficients Beta	Std. Error	Sig.
Age	−0.013	0.105	0.892
Gender	−0.153	1.831	0.118
Education	−0.120	0.781	0.250
Living arrangements	−0.076	1.804	0.442
Insurance	−0.749	6.009	0.901
Number of chronic conditions	**−0.228**	0.625	**0.033**
Knowledge of COVID-19 (low to high)	**0.264**	2.298	**0.010**
Perceived Threat of COVID-19 (less to high)	**0.306**	0.412	**0.004**
Attitudes toward COVID-19 vaccination	−0.112	1.156	0.278
Food environment	−0.046	0.860	0.639
Food insecurity	**−0.204**	0.848	**0.042**
Significance	0.005		
R-Square	0.245		

Note: Dependent Variable: HEI. Bold indicates significant at *p* < 0.05.

## Data Availability

Personal identification details of the participants were separated from the completed questionnaires. The data were stored in a locked room at the Charles R. Drew University of Medicine and Science. No information relating to identifiable individuals was disseminated at all. The data sets used and analyzed in the current study are available from the corresponding author for collaborative studies.

## References

[1] CDC (2019). Health of Black or African American Non-Hispanic Population. https://www.cdc.gov/nchs/fastats/black-health.htm.

[2] Yu D., Sonderman J., Buchowski M.S., Mclaughlin J.K., Shu X.-O., Steinwandel M., Signorello L.B., Zhang X., Hargreaves M.K., Blot W.J. (2015). Healthy Eating and Risks of Total and Cause-Specific Death among Low-Income Populations of African-Americans and Other Adults in the Southeastern United States: A Prospective Cohort Study. PLoS Med..

[3] Wang Y., Chen X. (2012). Between-Group Differences in Nutrition- and Health-Related Psychosocial Factors among US Adults and Their Associations with Diet, Exercise, and Weight Status. J. Acad. Nutr. Diet..

[4] Vergis S., Schiffer L., White T., Mcleod A., Khudeira N., Demott A., Fitzgibbon M., Hughes S., Tussing-Humphreys L. (2018). Diet Quality and Nutrient Intake of Urban Overweight and Obese Primarily African American Older Adults with Osteoarthritis. Nutrients.

[5] Kibe L.W., Bazargan M. (2022). Fruit and Vegetable Intake Among Older African American and Hispanic Adults with Cardiovascular Risk Factors. Gerontol. Geriatr. Med..

[6] Deierlein A.L., Morland K.B., Scanlin K., Wong S., Spark A. (2014). Diet Quality of Urban Older Adults Age 60 to 99 Years: The Cardiovascular Health of Seniors and Built Environment Study. J. Acad. Nutr. Diet..

[7] Hsiao P.Y., Mitchell D.C., Coffman D.L., Allman R.M., Locher J.L., Sawyer P., Jensen G.L., Hartman T.J. (2013). Dietary patterns and diet quality among diverse older adults: The University of Alabama at Birmingham Study of Aging. J. Nutr. Health Aging.

[8] Ning L., Niu J., Bi X., Yang C., Liu Z., Wu Q., Ning N., Liang L., Liu A., Hao Y. (2020). The impacts of knowledge, risk perception, emotion and information on citizens’ protective behaviors during the outbreak of COVID-19: A cross-sectional study in China. BMC Public Health.

[9] Sauer M.A., Truelove S., Gerste A.K., Limaye R.J. (2021). A Failure to Communicate? How Public Messaging Has Strained the COVID-19 Response in the United States. Health Secur..

[10] Dubé È., Labbé F., Malo B., Pelletier C. (2022). Public health communication during the COVID-19 pandemic: Perspectives of communication specialists, healthcare professionals, and community members in Quebec, Canada. Can. J. Public Health.

[11] Su Z., Zhang H., McDonnell D., Ahmad J., Cheshmehzangi A., Yuan C. (2022). Crisis communication strategies for health officials. Front. Public Health.

[12] Anker A.E., Feeley T.H., McCracken B., Lagoe C.A. (2016). Measuring the Effectiveness of Mass-Mediated Health Campaigns Through Meta-Analysis. J. Health Commun..

[13] Bogart L.M., Ojikutu B.O., Tyagi K., Klein D.J., Mutchler M.G., Dong L., Lawrence S.J., Thomas D.R., Kellman S. (2021). COVID-19 related medical mistrust, health impacts, and potential vaccine hesitancy among Black Americans living with HIV. J. Acquir. Immune Defic. Syndr..

[14] Bazargan M., Cobb S., Assari S. (2021). Discrimination and medical mistrust in a racially and ethnically diverse sample of California adults. Ann. Fam. Med..

[15] Mayasari N.R., Ho D.K.N., Lundy D.J., Skalny A.V., Tinkov A.A., Teng I.C., Wu M.C., Faradina A., Mohammed A.Z.M., Park J.M. (2020). Impacts of the COVID-19 Pandemic on Food Security and Diet-Related Lifestyle Behaviors: An Analytical Study of Google Trends-Based Query Volumes. Nutrients.

[16] Ammar A., Brach M., Trabelsi K., Chtourou H., Boukhris O., Masmoudi L., Bouaziz B., Bentlage E., How D., Ahmed M. (2020). Effects of COVID-19 Home Confinement on Eating Behaviour and Physical Activity: Results of the ECLB-COVID19 International Online Survey. Nutrients.

[17] Marty L., de Lauzon-Guillain B., Labesse M., Nicklaus S. (2021). Food choice motives and the nutritional quality of diet during the COVID-19 lockdown in France. Appetite.

[18] Martinez-Ferran M., de la Guia-Galipienso F., Sanchis-Gomar F., Pareja-Galeano H. (2020). Metabolic Impacts of Confinement during the COVID-19 Pandemic Due to Modified Diet and Physical Activity Habits. Nutrients.

[19] Nagata J.M., Seligman H.K., Weiser S.D. (2021). Perspective: The Convergence of Coronavirus Disease 2019 (COVID-19) and Food Insecurity in the United States. Adv. Nutr..

[20] Niles M.T., Bertmann F., Belarmino E.H., Wentworth T., Biehl E., Nef R. (2020). The Early Food Insecurity Impacts of COVID-19. Nutrients.

[21] Higashi R.T., Sood A., Conrado A.B., Shahan K.L., Leonard T., Pruitt S.L. (2022). Experiences of increased food insecurity, economic and psychological distress during the COVID-19 pandemic among Supplemental Nutrition Assistance Program-enrolled food pantry clients. Public Health Nutr..

[22] Parekh N., Ali S.H., O’Connor J., Tozan Y., M J.A., Capasso A., Foreman J., DiClemente R.J. (2021). Food insecurity among households with children during the COVID-19 pandemic: Results from a study among social media users across the United States. Nutr. J..

[23] Choi S.L., Men F. (2021). Food insecurity associated with higher COVID-19 infection in households with older adults. Public Health.

[24] Dubowitz T., Dastidar M.G., Troxel W.M., Beckman R., Nugroho A., Siddiqi S., Cantor J., Baird M., Richardson A.S., Hunter G.P. (2021). Food Insecurity in a Low-Income, Predominantly African American Cohort Following the COVID-19 Pandemic. Am. J. Public Health.

[25] Malek Rivan N.F., Yahya H.M., Shahar S., Ajit Singh D.K., Ibrahim N., Mat Ludin A.F., Mohamed Sakian N.I., Mahadzir H., Subramaniam P., Kamaruddin M.Z.A. (2021). The Impact of Poor Nutrient Intakes and Food Insecurity on the Psychological Distress among Community-Dwelling Middle-Aged and Older Adults during the COVID-19 Pandemic. Nutrients.

[26] Jackson A.M., Weaver R.H., Iniguez A., Lanigan J. (2022). A lifespan perspective of structural and perceived social relationships, food insecurity, and dietary behaviors during the COVID-19 pandemic. Appetite.

[27] Ankuda C.K., Fogel J., Kelley A.S., Byhoff E. (2021). Patterns of Material Hardship and Food Insecurity Among Older Adults During the COVID-19 Pandemic. J. Gen. Intern. Med..

[28] Ekwegh T., Cobb S., Adinkrah E.K., Vargas R., Kibe L.W., Sanchez H., Waller J., Ameli H., Bazargan M. (2023). Factors Associated with Telehealth Utilization among Older African Americans in South Los Angeles during the COVID-19 Pandemic. Int. J. Environ. Res. Public Health.

[29] Adinkrah E.K., Cobb S., Bazargan M. (2023). Delayed Medical Care of Underserved Middle-Aged and Older African Americans with Chronic Disease during COVID-19 Pandemic. Healthcare.

[30] Mujahid M.S., Diez Roux A.V., Morenoff J.D., Raghunathan T. (2007). Assessing the measurement properties of neighborhood scales: From psychometrics to ecometrics. Am. J. Epidemiol..

[31] Subar A.F., Kipnis V., Troiano R.P., Midthune D., Schoeller D.A., Bingham S., Sharbaugh C.O., Trabulsi J., Runswick S., Ballard-Barbash R. (2003). Using intake biomarkers to evaluate the extent of dietary misreporting in a large sample of adults: The OPEN study. Am. J. Epidemiol..

[32] Subar A.F., Thompson F.E., Kipnis V., Midthune D., Hurwitz P., McNutt S., McIntosh A., Rosenfeld S. (2001). Comparative validation of the Block, Willett, and National Cancer Institute food frequency questionnaires: The Eating at America’s Table Study. Am. J. Epidemiol..

[33] Diet History Questionnaire III (DHQ III). https://epi.grants.cancer.gov/dhq3.

[34] Blumberg S.J., Bialostosky K., Hamilton W.L., Briefel R.R. (1999). The effectiveness of a short form of the Household Food Security Scale. Am. J. Public Health.

[35] NIH (2020). PhenX Toolkit: COVID-19 Protocol Library. https://www.phenxtoolkit.org/.

[36] Krzyzanowski M.C., Terry I., Williams D., West P., Gridley L.N., Hamilton C.M. (2021). The PhenX Toolkit: Establishing standard measures for COVID-19 research. Curr. Protoc..

[37] Cox L.A., Hwang S., Haines J., Ramos E.M., McCarty C.A., Marazita M.L., Engle M.L., Hendershot T., Pan H., Hamilton C.M. (2021). Using the PhenX Toolkit to Select Standard Measurement Protocols for Your Research Study. Curr. Protoc..

[38] Bickel G., Nord M., Price C., Hamilton W., Cook J. Guide to Measuring Household Food Security. 2000, Revised. https://nhis.ipums.org/nhis/resources/FSGuide.pdf.

[39] Ellison B., McFadden B., Rickard B.J., Wilson N.L. (2021). Examining food purchase behavior and food values during the COVID-19 pandemic. Appl. Econ. Perspect. Policy.

[40] Ellison-Barnes A., Moran A., Linton S., Chaubal M., Missler M., Evan Pollack C. (2021). Limited Technology Access Among Residents of Affordable Senior Housing During the COVID-19 Pandemic. J. Appl. Gerontol..

[41] Trude A.C.B., Lowery C.M., Ali S.H., Vedovato G.M. (2022). An equity-oriented systematic review of online grocery shopping among low-income populations: Implications for policy and research. Nutr. Rev..

[42] Palmer F., Jung S.E., Shahan M.K., Ellis A. (2021). P66 Understanding How the COVID-19 Pandemic Influenced Older Adults’ Grocery Shopping Habits. J. Nutr. Educ. Behav..

[43] Murphy B., Benson T., Mccloat A., Mooney E., Elliott C., Dean M., Lavelle F. (2020). Changes in Consumers’ Food Practices during the COVID-19 Lockdown, Implications for Diet Quality and the Food System: A Cross-Continental Comparison. Nutrients.

[44] Boyacι-Gündüz C.P., Ibrahim S.A., Wei O.C., Galanakis C.M. (2021). Transformation of the Food Sector: Security and Resilience during the COVID-19 Pandemic. Foods.

[45] Jessica F.U. ‘I Felt Like I Failed’: Inflation Puts Healthy Food out of Reach for Millions of Americans. https://www.theguardian.com/environment/2022/sep/30/inflation-healthy-food-eating-america?utm_source=Dornsife+School+of+Public+Health+Communications&utm_campaign=cdb940349e-EMAIL_CAMPAIGN_2018_07_30_02_14_COPY_02&utm_medium=email&utm_term=0_fa9e5b0e03-cdb940349e-302144008&mc_cid=cdb940349e&mc_eid=3045c9f514.

[46] Duran-Aguero S., Vinueza-Veloz M.F., Gonzalez-Medina G., Carpio-Arias V., Rios-Castillo I., Cavagnari B.M., Nava-Gonzalez E.J., Camacho-Lopez S., Cordon-Arrivillaga K., Nunez-Martinez B. (2022). Psychological factors of diet quality among rural populations of Latin America during the COVID-19 pandemic: A cross-sectional study. Rural Remote Health.

[47] Iranmanesh M., Ghobakhloo M., Nilsashi M., Tseng M.-L., Senali M.G., Abbasi G.A. (2022). Impacts of the COVID-19 pandemic on household food waste behaviour: A systematic review. Appetite.

[48] Wang J., Rao N., Han B. (2021). Pathways Improving Compliance with Preventive Behaviors during the Remission Period of the COVID-19 Pandemic. Int. J. Environ. Res. Public Health.

[49] Harper C.A., Satchell L.P., Fido D., Latzman R.D. (2021). Functional Fear Predicts Public Health Compliance in the COVID-19 Pandemic. Int. J. Ment. Health Addict..

[50] Lee M., Kang B.A., You M. (2021). Knowledge, attitudes, and practices (KAP) toward COVID-19: A cross-sectional study in South Korea. BMC Public Health.

[51] Lockyer S. (2020). Effects of diets, foods and nutrients on immunity: Implications for COVID-19?. Nutr. Bull..

[52] Messina G., Polito R., Monda V., Cipolloni L., Di Nunno N., Di Mizio G., Murabito P., Carotenuto M., Messina A., Pisanelli D. (2020). Functional Role of Dietary Intervention to Improve the Outcome of COVID-19: A Hypothesis of Work. Int. J. Mol. Sci..

[53] Galmes S., Serra F., Palou A. (2020). Current State of Evidence: Influence of Nutritional and Nutrigenetic Factors on Immunity in the COVID-19 Pandemic Framework. Nutrients.

[54] Morales D.X., Morales S.A., Beltran T.F. (2021). Racial/ethnic disparities in household food insecurity during the COVID-19 pandemic: A nationally representative study. J. Racial Ethn. Health Disparities.

[55] Bowman S.A. (2009). Socioeconomic characteristics, dietary and lifestyle patterns, and health and weight status of older adults in NHANES, 1999–2002: A comparison of Caucasians and African Americans. J. Nutr. Elder..

[56] Chatters L.M., Taylor H.O., Taylor R.J. (2020). Older Black Americans During COVID-19: Race and Age Double Jeopardy. Health Educ. Behav..

